# Populational genomic insights of *Paraclostridium bifermentans* as an emerging human pathogen

**DOI:** 10.3389/fmicb.2023.1293206

**Published:** 2023-11-09

**Authors:** Xunchao Cai, Yao Peng, Gongli Yang, Lijuan Feng, Xiaojuan Tian, Ping Huang, Yanping Mao, Long Xu

**Affiliations:** ^1^Department of Gastroenterology and Hepatology, Shenzhen University General Hospital, Shenzhen University, Shenzhen, Guangdong, China; ^2^Marshall Laboratory of Biomedical Engineering, Shenzhen University, Shenzhen, Guangdong, China; ^3^College of Chemistry and Environmental Engineering, Shenzhen University, Shenzhen, Guangdong, China

**Keywords:** emerging pathogen, clostridial toxins, comparative genomics, *Paraclostridium bifermentans*, virulence factors, conserved plasmids

## Abstract

*Paraclostridium bifermentans* (P.b) is an emerging human pathogen that is phylogenomically close to *Paeniclostridium sordellii* (P.s), while their populational genomic features and virulence capacity remain understudied. Here, we performed comparative genomic analyses of P.b and compared their pan-genomic features and virulence coding profiles to those of P.s. Our results revealed that P.b has a more plastic pangenome, a larger genome size, and a higher GC content than P.s. Interestingly, the P.b and P.s share similar core-genomic functions, but P.b encodes more functions in nutrient metabolism and energy conversion and fewer functions in host defense in their accessory-genomes. The P.b may initiate extracellular infection processes similar to those of P.s and *Clostridium perfringens* by encoding three toxin homologs (i.e., microbial collagenase, thiol-activated cytolysin, phospholipase C, which are involved in extracellular matrices degradation and membrane damaging) in their core-genomes. However, P.b is less toxic than the P.s by encoding fewer secretion toxins in the core-genome and fewer lethal toxins in the accessory-genome. Notably, P.b carries more toxins genes in their accessory-genomes, particularly those of plasmid origin. Moreover, three within-species and highly conserved plasmid groups, encoding virulence, gene acquisition, and adaptation, were carried by 25–33% of P.b strains and clustered by isolation source rather than geography. This study characterized the pan-genomic virulence features of P.b for the first time, and revealed that *P. bifermentans* is an emerging pathogen that can threaten human health in many aspects, emphasizing the importance of phenotypic and genomic characterizations of *in situ* clinical isolates.

## Highlights

– P.b and P.s are hypothesized have similar mechanisms to initiate infections.
– P.b is probably less toxic than P.s by encoding fewer lethal toxins in the pangenome.
– Within-species-conserved and environmental-coevolved plasmids were identified in P.b.

## Introduction

Clostridia are bacteria belonging to the phylum *Firmicutes*, which are composed of a broad spectrum of Gram-positive (mostly), low GC content, spore-forming, and anaerobic bacilli (Orrell and Melnyk, 2021). Several genera of clostridia can cause mild to life-threatening diseases in both humans and animals, including tetanus and botulism, uterine infections, histotoxic infections and enteric diseases, by producing an array of protein toxins (Revitt-Mills et al., 2019; Zerrouki et al., 2021). Among the pathogenic clostridia, those grouped within clostridial cluster XI, such as *Clostridioides difficile* and *Paeniclostridium sordellii*, are particularly prevalent (Ezaki, 2009; Moore and Lacey, 2019). *C. difficile*, in particular, has attracted broad research efforts due to its clinical importance, and extensive studies have been conducted to clarify its virulence, strain diversity, transmission, and evolution at both the genomic and phenotypic levels (Knight et al., 2017). However, research on other clostridial cluster XI species, such as *Paraclostridium bifermentans*, whose clinical cases are uncommon, and has few genomic sequences publicly available or in most cases does not cause lethal diseases, is limited.

*P*. *bifermentans* is one of the two validly published species under the genus *Paraclostridium*, ubiquitously residing in various mesophilic conditions including soil, marine environments, polluted waters, and human bodies (Jyothsna et al., 2016). Our recent study has revealed that the well-characterized pathogen *P. sordellii* is phylogenomically closer to *P*. *bifermentans* than other pathogenic members within cluster XI (Zhao et al., 2022). *P*. *bifermentans* has traditionally been recognized as a human commensal, since it is usually nonpathogenic unless coexisting with *C. perfringens* (Weinberg and Séguin, 1918). However, a growing number of cases have been reported in clinical settings, demonstrating its ability to cause various human infections, such as brain abscess, lymphadenitis, necrotizing endometritis, joint infection, empyema, and endocarditis, particularly with the development of advanced diagnostic methods (Edagiz et al., 2015; Hale et al., 2016; Biswas et al., 2018; Barrett et al., 2020). Moreover, *P. bifermentans* PAGU1678^T^ was reported to exacerbate the pathological conditions of a dextran sulfate sodium-induced (DSS-induced) colitis mouse model in a recent study (Kutsuna et al., 2019). These findings have led us to appreciate that *P. bifermentans* has emerged as a pathogen in humans under specific conditions. While current observations suggest that infections caused by *P. bifermentans* are non-lethal, the possibility of high toxic strains cannot be excluded with such a limited number of reported cases.

As a member of clostridial cluster XI that is phylogenomically close to *P. bifermentans*, *P. sordellii* is commonly found in the rectal or vaginal tract of 3–4% of women, with the majority of carriers remaining asymptomatic (Aldape et al., 2016; Chong et al., 2016). Nevertheless, when pathogenic *P. sordellii* infections occur, they can rapidly progress and are associated with high mortality rates (~70%) due to the production of the lethal toxin protein TcsL, a member of the large clostridial toxin (LCT) family (Lee et al., 2020). It is worth noting that *C. difficile* also produces a toxin of the LCT family, namely TcdB, sharing 90% sequence identity with TcsL, which a major virulence factor responsible for *C. difficile* infections (Chen et al., 2018). In addition, clostridial species may also encode other potent virulence factors such as pore-forming cytotoxins, phospholipases, and metalloproteases that mediate their infections, many of which are encoded on extrachromosomal virulence plasmids (Revitt-Mills et al., 2019). Up to date, the virulence factor coding capability of *P. bifermentans* still remains largely unexplored, and as a result, our understanding of its pathogenesis and potential to cause lethal infections is limited.

In this study, we obtained the whole genome sequences of *P. bifermentans* and *P. sordellii* from the NCBI genome database. We analyzed the phylogenetic relationship between the two species by constructing a phylogenomic tree and performing whole-genome-sequence-based average nucleotide identity (ANI) analysis. Furthermore, we annotated and compared the pangenome and coregenome features of the two species, including general genomic characteristics such as genomic size, GC content, and coding density, as well as global gene functions and virulence factor coding profiles. Additionally, we explored the extrachromosomal toxin coding capacity of *P. bifermentans* for the first time by predicting, annotating, and grouping the virulence plasmid sequences based on sequence identity. The toxicity potential and pathogenesis of *P. bifermentans* were then proposed and discussed in detail.

## Materials and methods

### Genome sequence collection and preprocessing

The whole genome sequence sets from *Paraclostridium* spp. (i.e., *P. bifermentans* and *P. benzoelyticum*) and *P. sordellii* were downloaded from the NCBI genome Refseq database. The isolation sources of each strain were collected from the metadata table downloaded from the Refseq database or by searching the literature. The genomic sequences were evaluated for completeness and contamination using CheckM v1.0.12 (Parks et al., 2015). The standard lineage workflow “lineage_wf” from CheckM was performed to summarize general sequence features such as completeness, contaminations, genomic size, GC contents, coding density, and predicted rRNAs. Genomic sequences with completeness less than 70% or contamination greater than 10% were eliminated from the sequence sets. A total of 27 *Paraclostridium* spp. genomic sequences comprising one *P. benzoelyticum* strain JC272 and 26 *P. bifermentans* strains, and 60 *P. sordellii* genomic sequences were finally collected for further analysis (Supplementary Table S1).

### Whole-genome-sequence-based phylogeny

Each genome sequence in the genome sets was annotated using Prokka v1.14.5 with default parameters. Aligned core and accessory genome sequences of the genome sets were extracted separately from the resultant gff files using the Roary v3.11.2 pangenome pipeline (Page et al., 2015). The phylogenomic tree of each alignment (i.e., core and accessory) was constructed using FastTree v2.1.11 with the generalized time-reversible model. The average nucleotide identity (ANI) between all the collected sequences was computed using fastANI v1.33 (Jain et al., 2018), and the genome distance (GD) was calculated using the phylonium v1.2 (Klötzl and Haubold, 2020). The tanglegram between the core-genome tree and accessory-genome tree was visualized using the dendextend v1.15.2 R package. The ANI matrix and GD matrix were visualized using the pheatmap v1.0.12 R package. Finally, the core-genome tree with general features was visualized using iTol v6 (Letunic and Bork, 2021).

### Pan-genomic features and functional genome annotation

Plasmid sequences in the genome sets were predicted using (1) PlasForest v1.2,^1^ a machine learning-based tool that uses sequence homology, and (2) BLAST-based method with an e-value lower than 1e^−5^ and a sequence similarity higher than 90% and query coverage higher than 50% by aligning to the documented plasmids from *Paraclostridium* spp., *P. sordellii* and *C. perfringens*, and (3) circular contigs with no *dnaA* gene and no rRNA coding sequences although not predicted as plasmids based on the sequence homology methods. Pangenome features of either *P. bifermentans* or *P. sordellii* were extracted using the Roary v3.11.2 pangenome pipeline from the gff files produced by Prokka v1.14.5. Subsequently, the extracted pangenome features were analyzed and visualized using the R package Pagoo v0.3.12 (Ferrés and Iraola, 2021). The core genome was defined as the set of CDSs that are present in over 95% of genomes. All other CDSs were classified as the accessory-genome, which was further subdivided into the cloud genome (i.e., those present in less than 5% of genomes) and the shell genome (i.e., those present in 5–95% of genomes). The functional genome annotation was conducted using eggNOG-mapper v2.1.5, which comprehensively annotated the Carbohydrate-Active enZYmes (CAZy), Cluster of Orthologous Groups (COG), and Kyoto Encyclopedia of Genes and Genomes (KEGG) (Cantalapiedra et al., 2021). Virulence factor coding genes, including toxin genes and antimicrobial resistant genes, in the pangenome were predicted using PathoFact v1.0 with the “complete” parameter (de Nies et al., 2021).

## Results

### General genomic features and phylogeny characterization of *Paraclostridium bifermentans*

**Figure 1:**
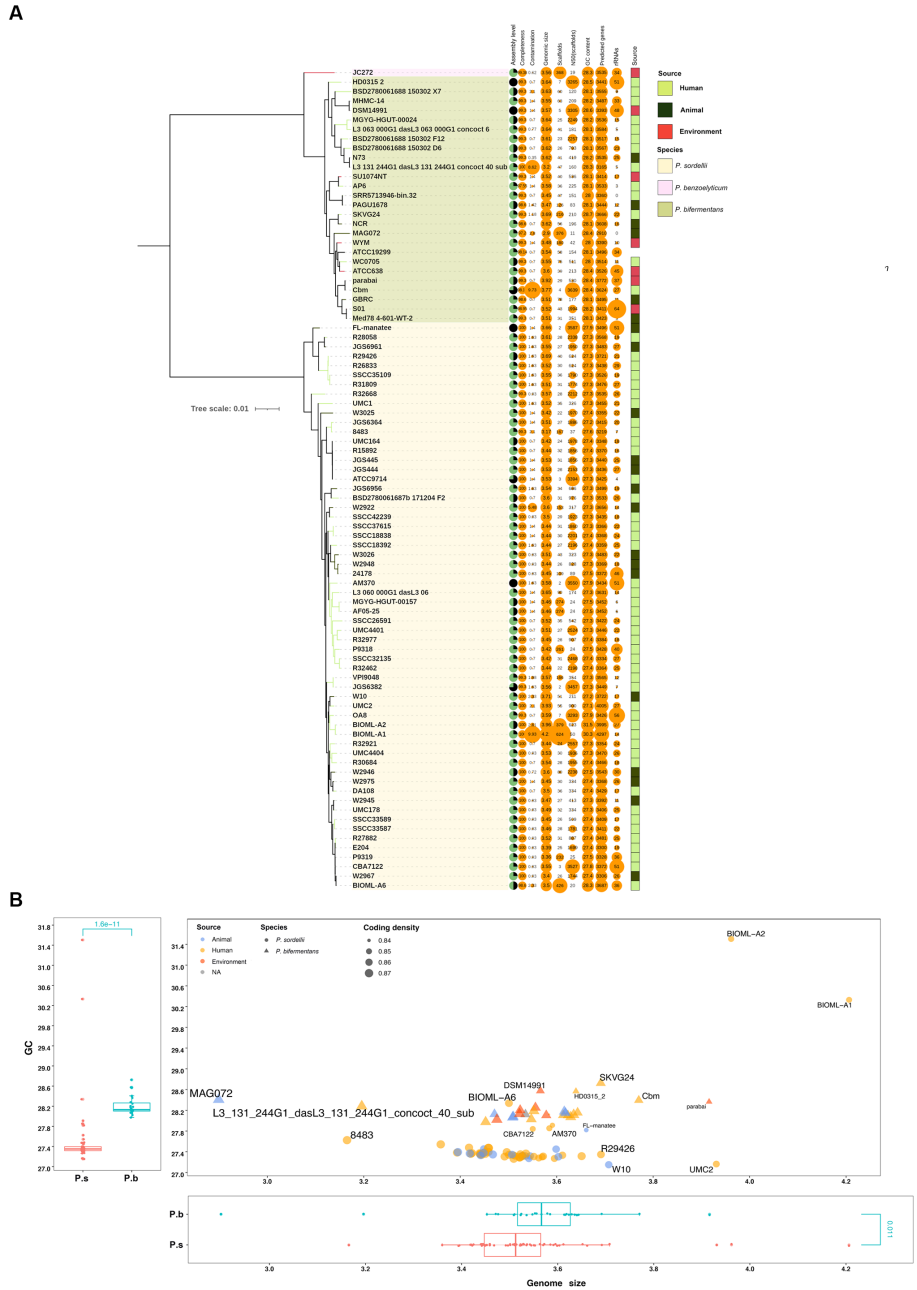
General genomic features of *P. bifermentans* and *P. sordellii*. **(A)** General genomic features and isolation sources of *P. bifermentans* and *P. sordellii* strains. The blank strip in the right means an undetermined isolation source. **(B)** Comparison of genomic size and GC content between *P. bifermentans* and *P. sordellii*. P.b and P. s represent *P. bifermentans* and *P. sordellii*, respectively. Differences in GC content and genomic size between P.b and P.s were analyzed using the Wilcox Test, with *p* < 0.05 considered statistically significant.

Prior to characterizing the genomic features, we carefully checked the isolation source of the strains. Interestingly, despite the ubiquitous presence of *P. sordellii* strains in the environment, none of the strains that had genomes sequenced are isolated from the environment, while 15 originated from animal sources and 45 from human sources. In contrast, strain *P. benzoelyticum* JC272 was isolated from the environment, and 6, 6, and 13 out of the 26 *P. bifermentans* strains were isolated from environmental, animal, and human sources, respectively (Figure 1A). Our analysis of the whole genome tree for both *P. bifermentans* (P.b) and *P. sordellii* (P.s) did not reveal any correlation between isolation source and within-species phylogeny, indicating that isolation source did not impact the phylogenetic relationships within these species (Figure 1A). Then, the collected genome assemblies were compared on assembly level, genome size, GC content, completeness, contamination, and predicted genes (Figure 1A). All of the genomes showed high completeness (> 99%), 4 out of the 87 genomes had medium contamination (5% < contamination <10%), and only 1 was assembled into more than 500 scaffolds. These features suggest that the genome set is of high quality. However, only 4 genomes were assembled at the completed level (2 from P.b and 2 from P.s), and 3 at the chromosomal level, and the others at the scaffold or contigs levels (Figure 1A). Of note, the rRNA sequence numbers showed high variability between strains within and between species. This is likely due to the limitations of short-read sequencing and assembly methods in accurately assembling the multicopy rRNA genes in bacteria. Analysis of completed genomes revealed that P.b and P.s possess a high number of rRNA genes, ranging from 48 to 51, which suggests a high protein synthesis capacity for these two species (Figure 1A). Additionally, these two species showed similar coding density, ranging from 0.84 ~ 0.87 (Figure 1B). The genome size and GC content of P.b are significantly higher than those of P.s (Figure 1B). For the following reasons, five genome sequences were excluded from further functional analyses: (1) they were identified as outliers in genome size and were assembled from metagenomes (i.e., MAG072, L3_131_244G1_dasL3_131_244G1_concoct_40_sub); (2) they were identified as belonging to a different species (i.e., strain JC272 of *P. benzoelyticum*); (3) they were identified as outliers in both genome size and GC content (i.e., BIOML-A1, and BIOML-A2) (Figure 1B).

**Figure 2:**
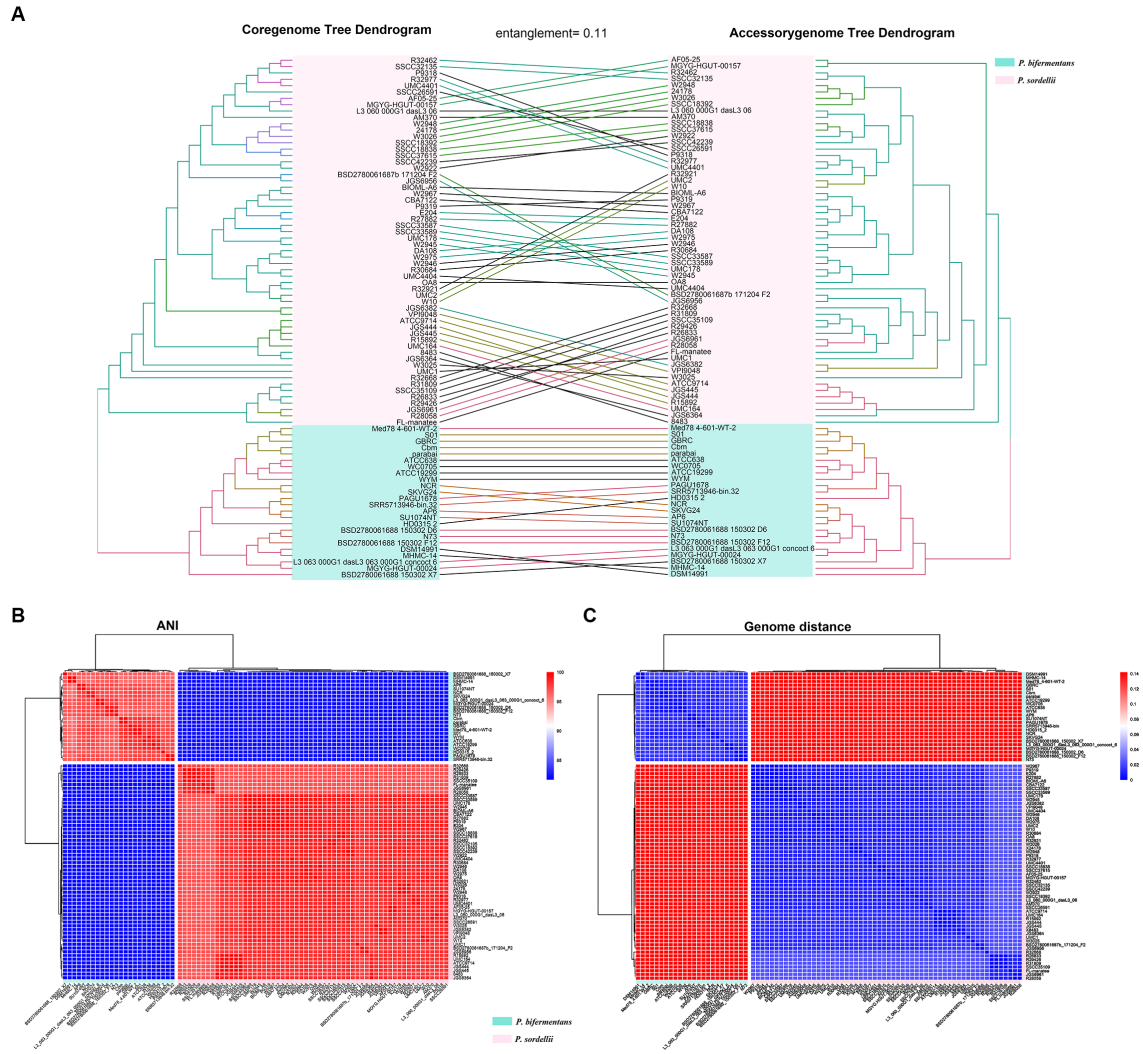
Phylogeny relationships between *P*. *bifermentans* and *P. sordellii* at the whole-genome sequence level. **(A)** Phylogenomic tree constructed using the core genome and accessory genome of the *P*. *bifermentans* spp. and *P. sordellii* genomes set. **(B)** ANI analysis between *P*. *bifermentans* and *P. sordellii* based on whole genome sequences. **(C)** Genome distances between *P*. *bifermentans* and *P. sordellii* based on whole-genome sequences.

As shown by the core-genome tree constructed from P.b and P.s, *P. bifermentans* is significantly divergent from *P. sordellii* (Supplementary Figure S1). The tangled dendrogram of the trees is displayed in Figure 2A. In detail, P.b and P.s are clearly separated by the topological structure of both the core-genomic and accessory-genomic tree, from which the tanglegram displayed highly consistence (entanglement = 0.11), underscoring a distinct divergence in phylogeny between P.b and P.s across both the core-genome and accessory-genome (Figure 2A). Furthermore, the heatmap and hierarchical clustering from ANI and GD further reveal the phylogenomic divergence of P.b and P.s at the species level (Figures 2B,C), highlighting a robust separation between the two species and substantiating the evolutionary divergence at both the core and accessory genomic levels.

### Pangenome characterization of *Paraclostridium bifermentans*

**Figure 3:**
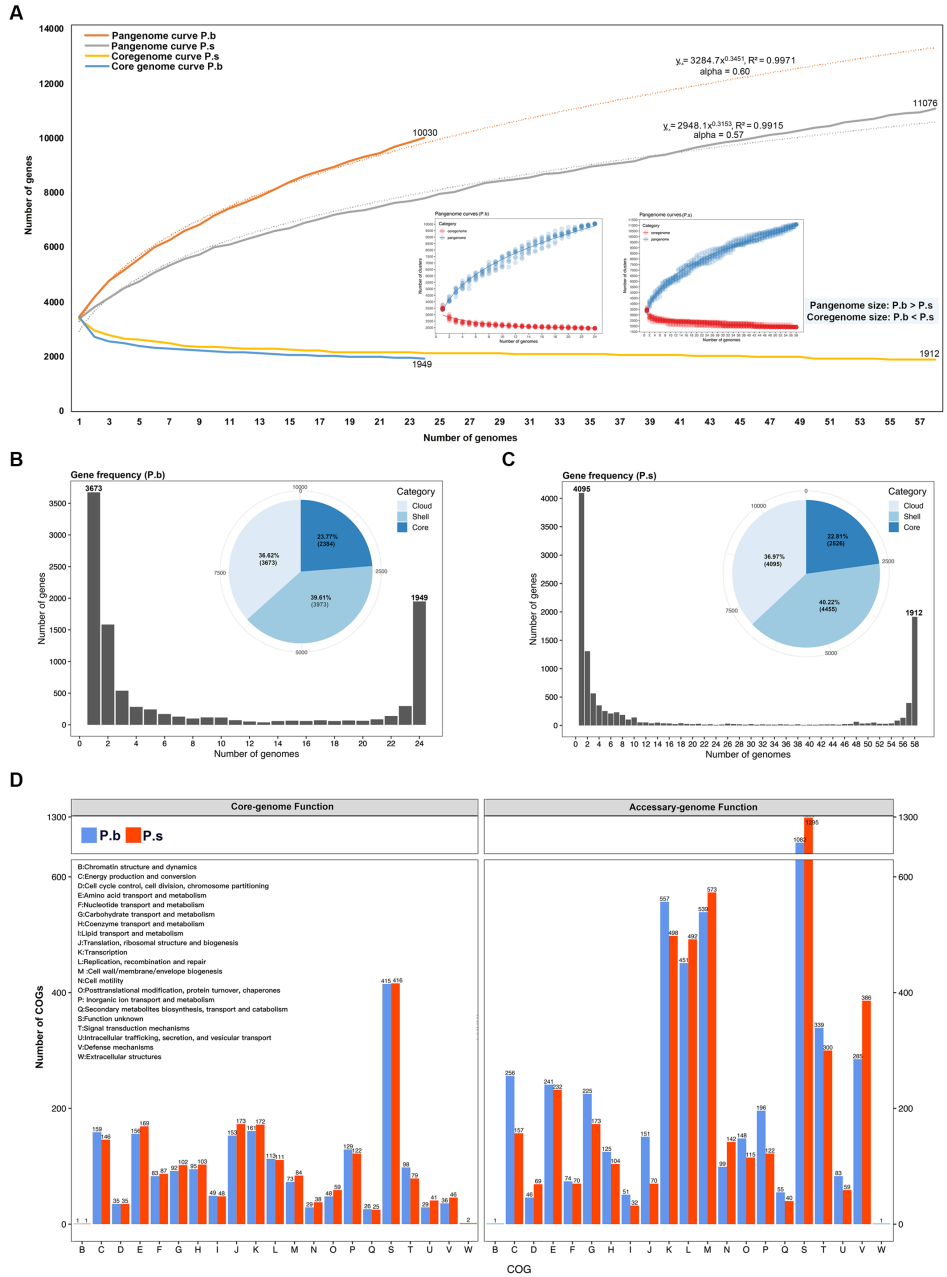
Comparison of pan-genomic features between *P. bifermentans* and *P. sordellii* strains. **(A)** Pan-genomic and core-genomic curves fitted using the positive-power model. **(B,C)** Gene frequency histogram and pangenome category pie-chart. **(D)** Pan-genomic functions displayed by clusters of orthologous groups (COGs).

To compare the coding capacity of P.b and P.s, we performed pangenome analyses using Roary. The pangenome of 24 *P. bifermentans* genomes and 58 *P. sordellii* genomes consisted of 10,030 and 11,076 orthologous genes, respectively. The pangenome curves for both species formed a “C” shape, and the alpha index of the heaps law models being lower than 1 (Figure 3A) (Tettelin et al., 2008), which revealed that both P.b and P.s carried open pangenomes (Figure 3A). The pangenome curves of P.b and P.s were best fitted for the positive-power model, with the formulas $yP.b=3284.7\chi 0.3451\;\left(R^{2}=0.9971\right)$ and $yP.s=2948.1\chi 0.3153\;\left(R^{2}=0.9915\right)$ , respectively, indicating that the pangenome of P.b is more plastic than that of P.s ( yP.b>yP.s ) (Figure 3A). Moreover, the core-genome size of P.b (2384) is comparatively smaller than that of the P.s (2526) (Figures 3B,C), which is further corroborated by the core-genome curves (Figure 3A). As the average genomic size of P.b is larger than that of P.s (Figure 2), the comparison in core-genome size between P.b and P.s further supported that P.b had a more plastic pangenome than that of P.s by carrying a greater number of accessory CDSs. The core-genome curve showed that 1949 orthologous genes are shared by all P.b strains, and 1912 orthologous genes are shared by all P.s strains, with the strain-specific genes in P.b and P.s are 3,673 and 4,095, respectively (Figures 3A–D). The core-genomes of P.b and P.s accounted for only 23.77% (2,384/10030) and 22.81% (2,526/11076) of the respective pangenome (Figures 3B,C). The core-genome-coded COG functions between P.b and P.s were similar in COG categories and counts, while the accessory-genome-coded COG functions showed high differences by COG counts (Figure 3D). P.b’s accessory-genome, for example, encoded more COG functions in nutrient metabolism, protein synthesis and energy conversion (e.g., “C Energy production and conversion,” “G Carbohydrate transport and metabolism,” “I Lipid transport and metabolism,” “J Translation, ribosomal structure and biogenesis,” “K Transcription,” “O Posttranslational modification, protein turnover, chaperones,” “P Inorganic ion transport and metabolism,” “T Signal transduction mechanisms” and “U Intracellular trafficking, secretion, and vesicular transport”), while that of P.s encoded more COG functions in cell cycle and host adaptation (e.g., “D Cell cycle control, cell division, chromosome partitioning,” “L Replication, recombination and repair,” “M Cell wall/membrane/envelope biogenesis,” “N Cell motility,” “S Function unknown” and “V Defense mechanisms”) (Figure 3D).

### Virulence genes carried by *Paraclostridium bifermentans*

**Figure 4:**
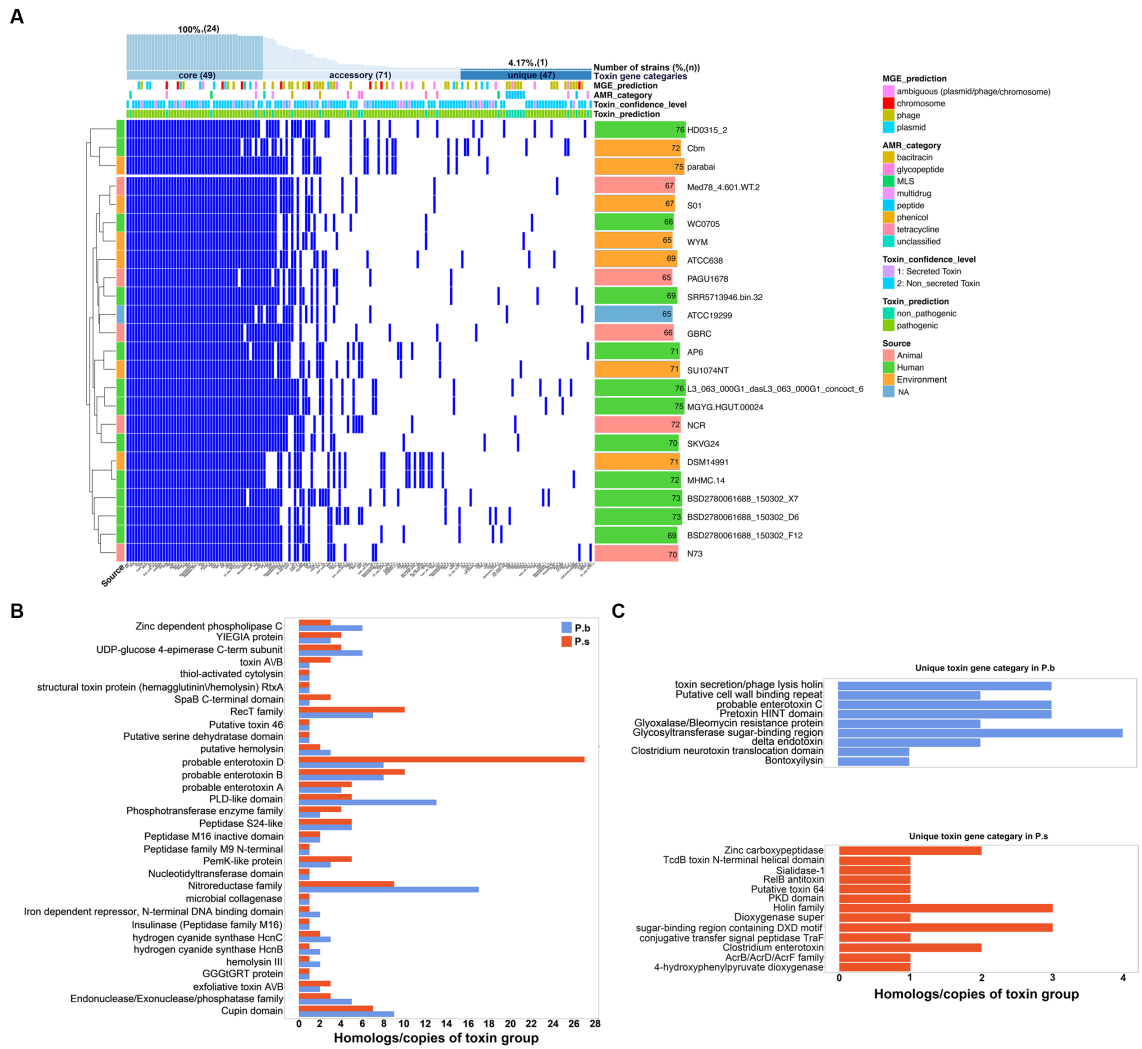
Characterization of the virulence factor coding capacity of *P. bifermentans*. **(A)** Heatmap showing the presence or absence of virulence factor coding genes in the pangenome of *P. bifermentans*. **(B,C)** The shared **(B)** or unique **(C)** virulence gene categories between *P. bifermentans* and *P. sordellii*.

We further explored the virulence factor coding capacity of P.b and P.s to compare their pathogenic potentials. The virulence gene profiles between P.b and P.s were divergent, enabling effective discrimination between the two species (Supplementary Figure S2a). Specifically, each P.s strain carried an average of 71 ± 3 virulence genes, while each P.b strain carried an average of 70 ± 4 virulence genes. A total of 176 and 167 virulence genes were carried by the pangenomes of P.s and P.b, respectively. Among them, 29.34% (49/167) and 31.25% (55/176) belonged to the core-genomes of P.b and P.s, respectively, accounting for approximately 70% of the virulence genes in each strain (Supplementary Figure S2B, Figure 4A). Furthermore, P.s carried 39 virulence genes encoding secreted toxins in the pangenome, and 14 of them were in the core-genome, whereas that of P.b was 28, and only 9 of them were in the core-genome. Since we had confirmed in this study that P.b carried a much opener pangenome than that of P.s (Figure 3A), we can deduct from the results here that the core-genome of P.b may encode less secreted-toxin genes than that of P.s, with the available P.b genomes increased. In addition, 44.91% (75/167) of the virulence genes in P.b were from MGEs, 19 of which are core genes, and over half of the MGE-related virulence genes (40/75) are phage-originated (Figure 4A). Similar findings were observed in P.s (Supplementary Figure S2B). The MGEs, especially the phage-originated MGEs, play vital roles in the expansion of the virulence gene pool in the pangenomes of P.b and P.s (Figure 4A, Supplementary Figure S2B). Regarding virulence gene families, most of them were shared by the pangenomes of P.b and P.s, but the gene counts varied. For example, the P.b pangenome coded more Zinc-dependent phospholipase C, UDP-glucose 4-epimerase C-term subunit, PLD-like domain, and Nitroreductase family virulence factors, while that of P.s coded more toxin A/B, SpaB C-terminal domain, and enterotoxin D virulence factors (Figure 4B). Additionally, there are several species-specific toxin gene families, such as enterotoxin C, pretoxin HINT domain, and Clostridium neurotoxin translocation domain in P.b, and Zinc carboxypeptidase, and Clostridium enterotoxin in P.s (Figure 4C). A detailed virulence gene co-occurrence network between P.b and P.s is shown in Supplementary Figure S3.

**Figure 5:**
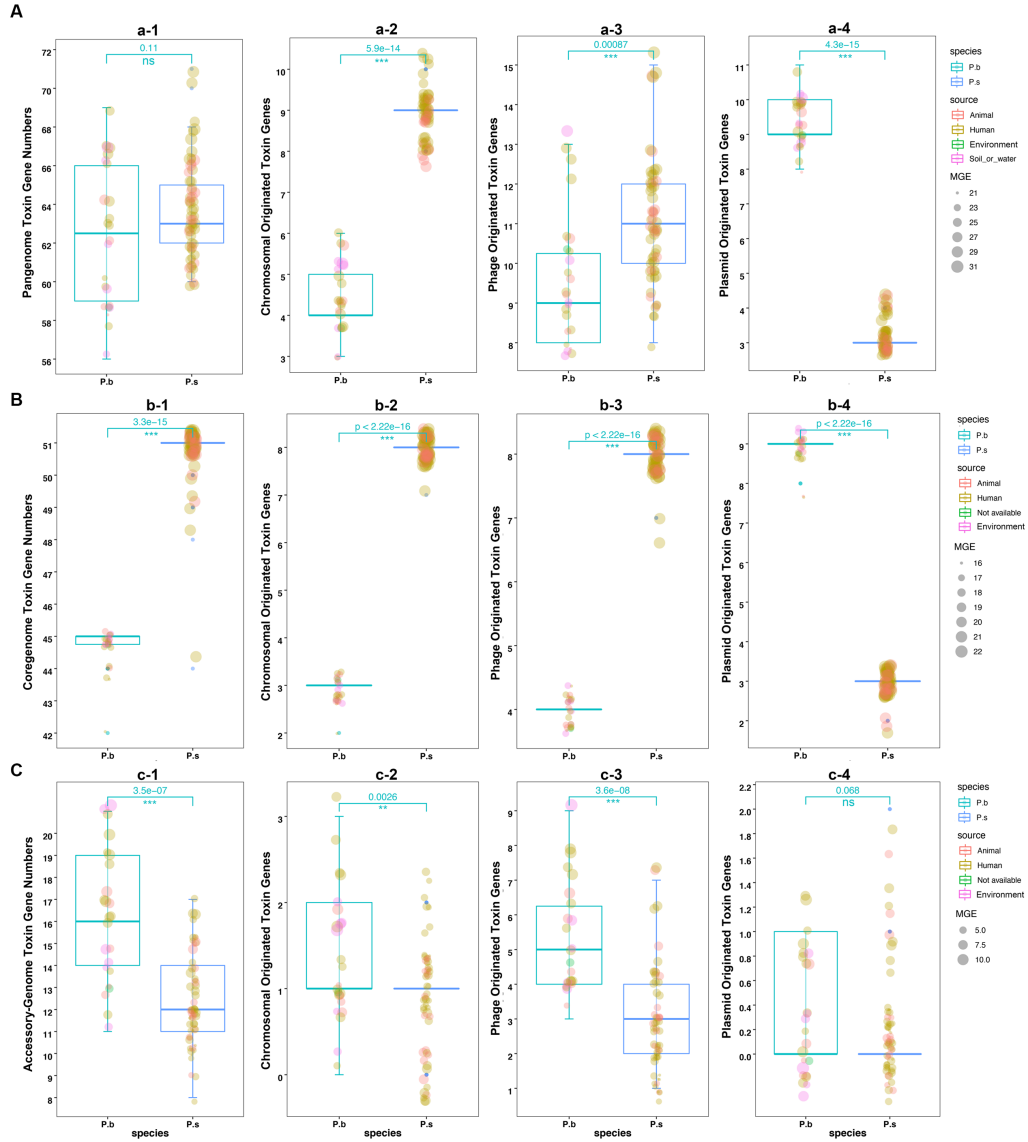
Comparison of the toxin genes in *P. bifermentans* and *P. sordellii* by sequence origination types. Toxin genes coded in pangenome **(A)**, coregenome **(B)**, and accessory-genome **(C)**. The subplots (e.g., a-2, a-3, a-4) indicate the toxin gene sequence type from the MGE prediction using PathoFact, and unclassified predictions were excluded from the plots. The Wilcox Test was used to compare the difference in toxin gene counts between the pangenome, coregenome, and accessory-genome, as well as different sequence-origination types, with a *p*-value <0.05 considered significant.

We further compared the toxin gene counts derived from different sequence types to estimate the virulence coding capacity of P.b and P.s. The results showed no significant difference in the pangenome-coded toxin gene counts between P.b and P.s. However, the coregenome-coded toxin genes in P.s were higher than those in P.b (Figures 5A–C). Moreover, both chromosomal- and phage-originated toxin gene counts in the pangenome or coregenome of P.s were higher than that in P.b (Figures 5A,B), which displayed an opposite pattern in the accessory-genome (Figure 5C). It is noteworthy that the plasmid-originated toxin genes were higher in both the pangenome and coregenome of P.b than in P.s (Figures 5A,B). As a result, the plasmid-originated sequences played a significant role in shaping the toxin gene profile of P.b’s coregenome, while the phage-originated sequences played a significant role in that of the accessory-genome (Figures 5B,C).

Furthermore, we compared the presence of the common reported clostridial toxin genes in P.b and P.s. The results showed that the pangenomes carried a total of eight toxin gene homologs, out of which five, namely *colA*, *pfo*, *plc*, *toxB/tcsL*, and *iap*, were shared by both species. The former three genes were core genes in both species that coded for proteins involved in host cell surface attachment and cell membrane damage, indicating that P.b and P.s may share similar mechanisms for initiating infections (Table 1). The *toxB/tcsL* and *iap* that encode host cell intracellular toxicity, which may lead to lethal and dermonecrotic effects, are presented more frequently in P.s than in P.b, indicating that the P.s is more toxic than the P.b (Table 1). The remaining three genes, namely *toxA/tcsH*, *plcB* and *cpe*, were specifically carried by P.s, coding functions of hemorrhagic toxin, pathogen’s vacuole escape, and cell pore-forming. Furthermore, the *plcB* gene was a core gene in P.s but absent in P.b (Table 1).

**Table 1:** Presence of typical clostridial toxin gene homologs in *P. bifermentans* and *P. sordellii.*

Toxin gene homologs^a^	Presence in P.b (%, *n*/*n*)	Presence in P.s (%, *n*/*n*)	Gene products	Possible virulence mechanisms
*colA*	100%, 24/24	100%, 58/58	Microbial collagenase	Extracellular matrices degradation
*pfo*	100%, 24/24	98.28%, 57/58	PFO (thiol-activated cytolysin)	Pore-formation
*plc*	95.83%, 23/24	100%, 58/58	PLC (Zinc dependent phospholipase C)	Membrane damage
*toxB/tcsL*	4.17%, 1/24	12.07%, 7/58	toxin B	Infiltration and destroying
*iap*	12.5%, 3/24	100%, 58/58	Probable enterotoxin	Actin cytoskeleton destroying
*toxA/tcsH*	0	12.07%, 7/58	toxin A	Cytoskeletal structure disruption
*plcB*	0	100%, 58/58	Peptidase M16 inactive domain	Pathogen’s vacuole escape mediation
*cpe*	0	3.45%, 2/58	Clostridium enterotoxin	Pore-formation

a Gene symbol was designated by using the Prokka and the gene function was predicted using the PathoFact.

### Virulence plasmid groups in *Paraclostridium bifermentans*

**Figure 6:**
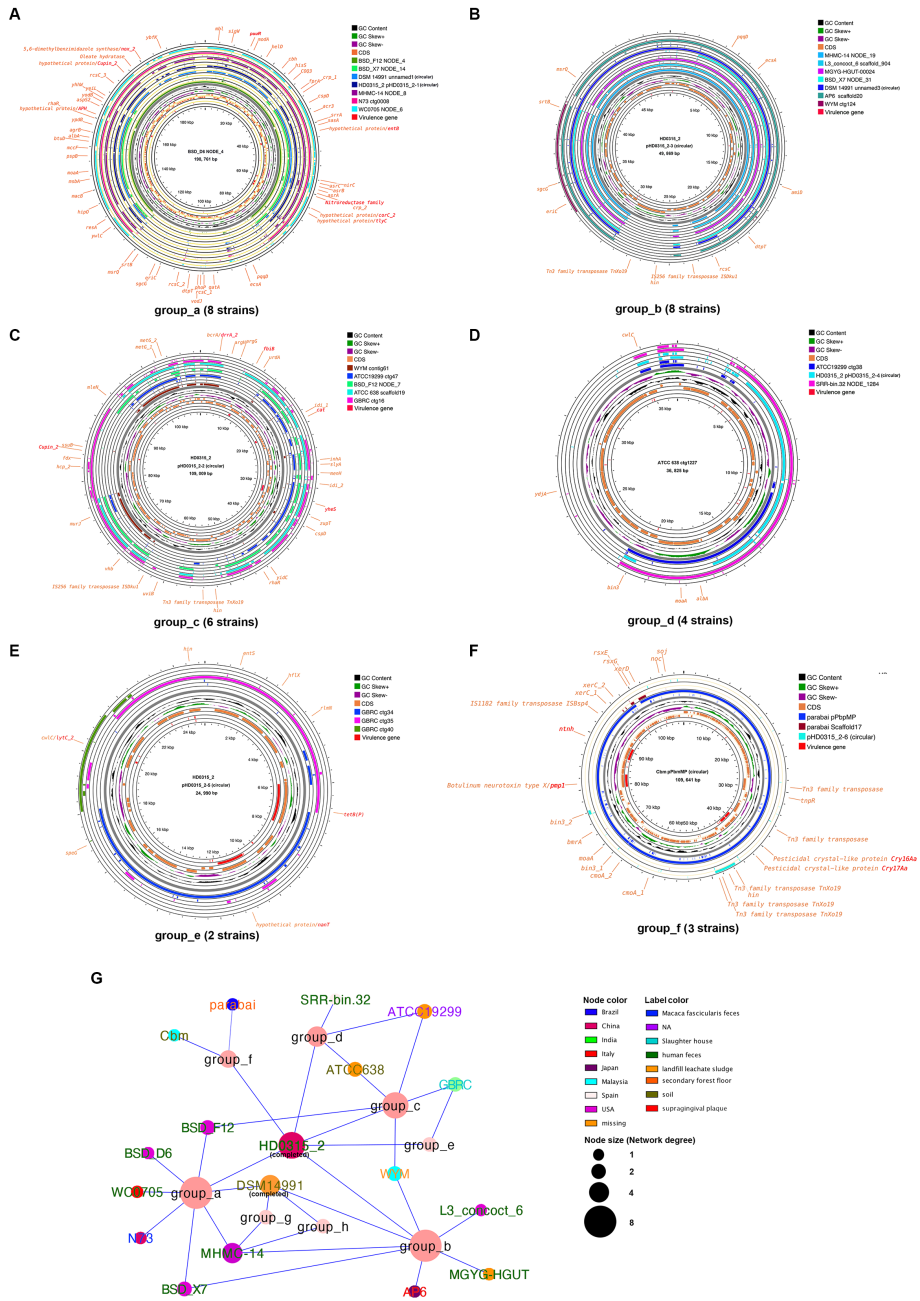
Representative plasmid groups in *P. bifermentans* and their coded functions. **(A–F)** Group_a to group_f plasmids in *P. bifermentans*. The longest plasmid sequence in each group was represented as the reference sequence. CDSs annotated as hypothetical proteins were not labeled in the plots. **(G)** Co-existence network of the conserved plasmid groups in P.b.

To elucidate the role of plasmids in the virulence of P. b, the plasmid sequences in the genomes of P.b were predicted and extracted using the methods described in the methods section. Plasmid pPbm14_8 from strain Cbm was depleted in further analyses because of that the pPbm14_8 was identified as a chromosome sequence by aligning to the NCBI-nt database (Supplementary Table S2). By aligning the reported circular plasmid sequences in P.b and P.s strains, we then found that all plasmids between species are divergent, while some of those are highly conserved within-species (Supplementary Figure S4a). Further analyses on the plasmid sequence similarity between *C. perfringens*, P.b and P.s also revealed high within-species conservation (Supplementary Figure S4b). Finally, eight conserved plasmid groups in P.b were identified, ranging from 25 kb to198 kb in length (Figures 6A–F, Supplementary Figure S5). Among them, group_a, group_b and group_c are the most conserved groups with a presence in 25–33.33% P.b. strains, which were carried by 8, 8 and 6 P.b strains, respectively (Figures 6A–C). Plasmids belonging to group_a are the longest and two of them (i.e., pHD0315_2–1 and DSM14991 unnamed1) were assembled at the completed level (Figure 6A). The longest plasmid in group_a is from strain BSD_D6, the NODE_4 in the draft genome, which is 198,761 bp, 40 kb longer than other plasmids in this group (Figure 6A). This group of plasmids encode about 150–180 CDSs, of which one third can be annotated, and is the best annotated plasmid group in P.b (Figure 6A). The reference plasmid in this group carries eight potential virulence genes, of which five (i.e., *Cupin_2*, *nitroreductase family*, *puuR*, *corC_2*, and *tlyC*) are core genes in P.b (Figures 4A, 6A). It is noteworthy that the plasmids in this group are essential for regulating bacterial growth and adaptation through the encoding of various functions related to organic sulfate reduction (*asrABC*, *nirC*), 5,6-dimethylbenzimidazole synthesis (*nox_2*), styrene degradation (*gatA*), aminoacyl-tRNA biosynthesis (*aspC*, *gatA*), etc. (Figure 6A). Additionally, this group of plasmids encode five virulence genes, two of which, namely *cat* and *drrA*_2, confer resistance to chloramphenicol and doxorubicin. Group_f represents a rare but typical virulence plasmid group in P.b that is exclusively carried by two strains, namely parabai and Cbm, isolated from anopheline-endemic areas in Malaysia and Brazil, respectively. This plasmid group encodes two pesticidal toxins (Cry16Aa and Cry17Aa) and one novel neurotoxin, which are flanked by Tn3 and IS1182 family transposases, respectively (Figure 6F). Notably, the Tn3 transposase is also encoded by other plasmid groups (e.g., group_b and group_c) (Figures 6B,C). While group_b and group_e plasmids are less annotated, some of the genes they carry, such as *pqqD*, *srtB* and *dtpT*, are related to pyrroloquinoline quinone synthesis (Ludueña et al., 2017), surface anchoring (Weiss et al., 2004), and salt stress protection (Wouters et al., 2005). Conversely, group_d, group_g and group_h plasmids are poorly annotated (Figure 6D, Supplementary Figures S5g,h). Interestingly, strains carrying plasmids clustered in the same conservation group were isolated from distant geographic locations (Figure 6G), which may be clustered by the isolation-source and have co-evolved with their habitats, as we found that the group_a and group_b plasmids are predominantly carried by strains isolated from feces (Figure 6G). Furthermore, strains with completed genome sequences (i.e., HD0315_2 and DSM14991) carried more plasmids than those with draft genome sequences. Unfortunately, over half of the strains with genome sequences are not isolated from clinical settings (Figure 6G).

## Discussion

As a species that is phylogenomically close to the pathogen P.s in clostridial XI, P.b is being recognized as an emerging human pathogen. Despite this, the strain-level diversity, toxicity, and genomic virulence capacity of P.b strains are yet understudied. This study reveals that P.b possesses a larger genome size with a higher GC content than P.s. Additionally, both P.b and P.s possess an open pangenome, with that of P.b being more plastic. While the gene function profiles of P.b and P.s are similar in the core-genomes, they differ significantly in the accessory genomes. P.s is more toxic than P.b, as it carries more toxin genes and encodes more secretion toxins in the core-genome and carries lethal toxin genes in the accessory genome. However, plasmid-originated toxin genes are more abundant in the pangenome or core-genome of P.b. Notably, plasmids were discovered to be species-conserved among P.b., P.s., and C.p. Furthermore, these plasmids were discovered to be clustered not by geographic location but by isolation source in P.b. This study also identified conserved Tn3 and IS1182 family transposase coding genes flanked by toxin coding genes in P.b, which may facilitate the acquisition and spread of virulence genes for P.b strains.

Previous studies have revealed that a larger genome and higher GC content elicit bacteria adaptation in more complex and varied environments (Lassalle et al., 2015). In the case of P.b, this species possesses a larger genomic size and higher GC content than P.s, which may confer an enhanced capacity to thrive across diverse environments (Figure 1). Our study revealed that P.b strains were isolated from a broader range of sources, despite the fact that the number of sequenced P.b genomes is currently fewer than those of P.s (Figure 1A). The versatility of P.b can be attributed to its adaptive capacity and genomic plasticity, which is primarily determined by the accessory genome. For instance, P.b strains have been utilized in various applications such as metal-contamination bioremediation (Neveling et al., 2022), antibiotic degradation (Fang et al., 2021) and organic compound conversion (Huang et al., 2022). In contrast, P.s strains, those that had genomes sequenced were all isolated from animal or human hosts (Figure 1A) and are commonly involved in infections (Zerrouki et al., 2021; Gonzalez-Astudillo et al., 2022), indicating that they may have adapted to these environments by encoding more host defense functions (Figure 3D). Nevertheless, it is important to note that the host adaptation capacity of P.b should not be underestimated, as the P.b carries a highly plastic pangenome, whereas the number of isolated P.b strains and sequenced genomes from clinical specimens is increasing.

P.b has been reported to be clustered into the clostridial cluster XI with *C. difficile* and P. s, and phylogenomically closer to the latter (Moore and Lacey, 2019; Zhao et al., 2022). The virulence of *C. difficile* and P. s is largely determined by their production of a wide array of toxins, although some non-toxin factors, such as degradative enzymes, surface-exposed proteins, and adhesion factors, also play a role (Revitt-Mills et al., 2019; Geier et al., 2021). We predicted for the first time the full-spectrum virulence gene profile of P.b, revealing a distinct virulence gene pool compared to that of P.s (Figure 4). P.s possesses a higher pathogenic potential than P.b, due to the encoding of more secreted toxins in the coregenome and more LCT toxins in the accessory genome (Figure 4). Although P.b infections are much rarer than P.s infections in clinical settings (Revitt-Mills et al., 2019), and no lethal infections have been reported, it is possible that lethal infections of P.b may occur in humans. This is supported by our identification of *toxB* and *iap* homologs in 4–12% of P.b genomes (Figure 4A).

It is worth noting that P. b and P. s may share similar extracellular infection mechanisms with C.p, as evidenced by the presence of three core toxin gene homologs (i.e., *colA*, *pfo* and *plc*) (Table 1), which have been reported to lead to extracellular matrix degradation, cell pore-formation and cell membrane damage in host cells (Popoff and Bouvet, 2009). Studies have demonstrated that these toxins can exhibit synergistic effects during the infections (Popoff and Bouvet, 2009; Popoff, 2016). Nevertheless, the intracellular cytotoxicity of P.b may be much lower than that of P.s, because only one toxin gene (i.e., *iap*), encoding actin cytoskeleton destroying functions, is present in 12.5% of the P.b strains compared to 100% present in P.s strains (Table 1). Additionally, P.s strains exclusively carried three toxin genes encoding cytoskeletal structure disruption, pathogen’s vacuole escaping and pore-formation, making them more lethal than P.b strains (Table 1). Further sequence similarity analysis using NCBI blastp revealed that the three core toxin protein homologs clustered by a species-conserved pattern and were divergent from that of C.p (Supplementary Figure S6). Previous research has revealed that the P.b PLC is weakly hemolytic and nonlethal compared to the C.p PLC, and the C-terminal of P.b PLC lacks two residues, Tyr331 and Phe334, that are present in the C.p PLC (Popoff and Bouvet, 2009). Moreover, varying degrees of pore-formation cytotoxicity of the PFO homologs have also been observed in *Bacillus*, *Listeria*, and *Streptococcus* (Hotze et al., 2013), contributing to pathogenesis through processes such as intracellular protein translocation (*S. pyogenes*), phagosomal escaping (*L. monocytogenes*), and macrophage escaping (C.p) (Portnoy et al., 1988; Madden et al., 2001; O'Brien and Melville, 2004). However, the specific effects of PFO and PLC homologs in P.b and P.s on cytotoxicity are yet to be explored. As a matter of fact, the P.b and P.s strains rarely cause human infections, from which we can deduce that the PLC and PFO homologs in them are less toxic than those found in C.p. However, wet-bench experiments are required to clarify the exact cytotoxicity of these toxin homologs in the future.

Many clostridial virulence factors, particularly lethal toxins, are encoded on large plasmids or other MGEs (e.g., bacteriophage, insert elements, and transposons). The mobile nature of these virulence genes facilitates their wide dissemination within the clostridial genera (Revitt-Mills et al., 2019). The lethal LCTs and the associated regulators in P.s (i.e., TcsL and TcsH) have been found encoded by the pCS1 family plasmids, which are conserved in at least seven strains (Vidor et al., 2018). In addition, all members of the pCS1 family encode a number of surface-exposed proteins (e.g., sortase enzyme (*srtB*)). Other plasmid families, such as pCS2, have also been identified in P.s, but they are poorly characterized (Couchman et al., 2015). Our study demonstrated that the number of plasmid-originated toxin genes in P.b is greater than that in P.s in the coregenome (Figure 5), and eight conserved plasmid groups were identified in P.b (Figure 6). Interestingly, no inter-species plasmid exchange between P.b, P.s, and C.p was observed, despite the fact that *C. perfringens* and P.b frequently co-existed under clinical circumstance (Supplementary Figure S4). Unlike P.s plasmids, none of the P.b plasmids coded for lethal toxins, whereas they play important roles in the virulence, adaptation, and metabolism of P.b. Particularly, group_a plasmids, which are present in one-third of P.b strains, carry virulence genes, as well as several organic metabolism and biosynthesis genes (Figure 6A). For instance, *corC* coded functions for magnesium and cobalt efflux (Huang et al., 2021); *tlyC* coded functions for phagosomal escape (Whitworth et al., 2005); *asrABC*-*nirC* cluster coded functions for microbial sulfidogenesis, which produces genotoxic hydrogen sulfide as a potential trigger of colorectal cancer (Czyzewski and Wang, 2012; Wolf et al., 2022). Of note, the rarely appearing group_f plasmids in P.b encoded two types of mosquito toxins (i.e., the Cry16Aa/ Cry17Aa pair, and the novel neurotoxin PMP1) that are carried only by two strains isolated from Malaysia and Brazil, which are geographically distant but populated with mosquito species, indicating a gene acquisition with insect–bacteria coevolution (Contreras et al., 2019). A similar pattern of plasmid coevolution within species was observed for group_a plasmids, which were mostly carried by strains isolated from human feces in different geographic locations (Figure 6). Moreover, the Tn3 and IS1182 family transposases flanked in the toxin genes in group_f plasmids were sporadically presented in other plasmid groups distinct form group_f, providing strong evidence that the transposases in P.b contribute greatly in broadening of its gene pool for virulence and adaptation (Figure 6A). Up to date, only twenties P.b strains have had their genomes sequenced, and *in situ* clinical isolates are rare. Therefore, more efforts should be made in the future to isolate *in situ* clinical strains and to complete their whole genome sequences to obtain more accurate population genomic characteristics of the plasmids.

## Conclusion

In conclusion, this study revealed that *P. bifermentans* carries a highly plastic pangenome as an emerging human pathogen. The core-genome of this species encodes at least three clostridial toxin homologs that are associated with extracellular matrix degradation and cell membrane damage, which can initiate extracellular infections. MGEs, particularly plasmids, have greatly contributed to the broadening of virulence, adaptation, and metabolism gene pools of this species. Although no lethally toxic strains in this species have been reported, the high prevalence of plasmids and transposons in the genomes highlights the potential risk for the emergence of lethal strains. Of note, this species may trigger colorectal cancer by producing genotoxic hydrogen sulfide in the gut tract. Inherent limitations arise in this study due to the absence of validation for specific clinically-related results through wet-bench experiments, and the scarcity of clinically-derived *P. bifermentans* genomes in public databases hampers extensive large-scale analyses. Nevertheless, this study significantly contributes to our understanding of the population genomics insights on phylogeny and pathogenesis associated with the emerging human pathogen *P. bifermentans*. The findings underscore the imperative for enhanced efforts in the isolation and sequencing of clinically-relevant *P. bifermentans* strains. Such endeavors are crucial to proactively mitigate the potential threats posed by these strains to human health.

## Data availability statement

The original contributions presented in the study are included in the article/Supplementary material, further inquiries can be directed to the corresponding authors.

## Author contributions

XC: Data curation, Funding acquisition, Writing – original draft. YP: Data curation, Writing – original draft. GY: Investigation, Methodology, Writing – review & editing. LF: Resources, Writing – review & editing. XT: Validation, Writing – review & editing. PH: Investigation, Writing – review & editing. YM: Conceptualization, Funding acquisition, Supervision, Writing – review & editing. LX: Conceptualization, Funding acquisition, Supervision, Writing – review & editing.

## Footnotes

^1^ https://github.com/leaemiliepradier/PlasForest
