# Supplementary material for: Populational genomic insights of Paraclostridium bifermentans as an emerging human pathogen
Source: Front Microbiol. 2023 Nov 9;14:1293206. doi: 10.3389/fmicb.2023.1293206 (PMC10665999; doi:10.3389/fmicb.2023.1293206)
Supplement: Supplementary file 2 [file Data_Sheet_1.pdf]

Populational Genomic insights of *Paraclostridium bifermentans* as an Emerging Human Pathogen

Xunchao Cai [a, b], Yao Peng [a], Gongli Yang [a], Lijuan Feng [a], Xiaojuan Tian [a], Ping Huang [a], Yanping Mao [c, *], Long Xu [a, b*]

[a] Department of Gastroenterology and Hepatology, Shenzhen University General Hospital, Shenzhen University, Shenzhen, Guangdong, 518071, China

[b] Marshall Laboratory of Biomedical Engineering, Shenzhen University, Shenzhen, Guangdong, 518071, China

[c] College of Chemistry and Environmental Engineering, Shenzhen University, Shenzhen, Guangdong, 518071, China

Running title: Populational Genomic Features of *P. bifermentans*

Correspondence:

Long Xu, Department of Gastroenterology and Hepatology, Shenzhen University General Hospital, Shenzhen University, 518071, Shenzhen, China. Email: longxu1012@szu.edu.cn; and Yanping Mao, College of Chemistry and Environmental Engineering, Shenzhen University, Shenzhen, Guangdong, 518071, China. Email: maoy@szu.edu.cn.

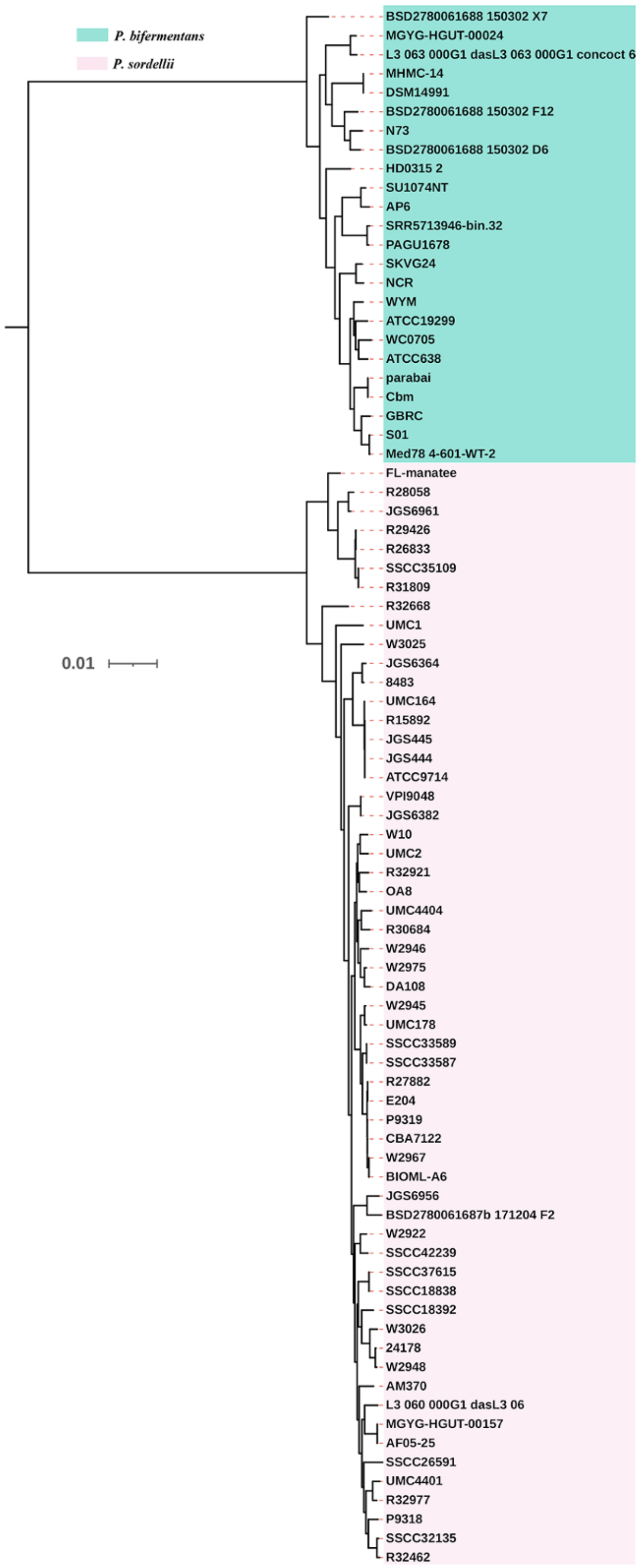

Figure S1 Coregenome tree of *P. sordellii* and *P. bifermentans*. The tree was rooted by the *Paraclostridium* clade.

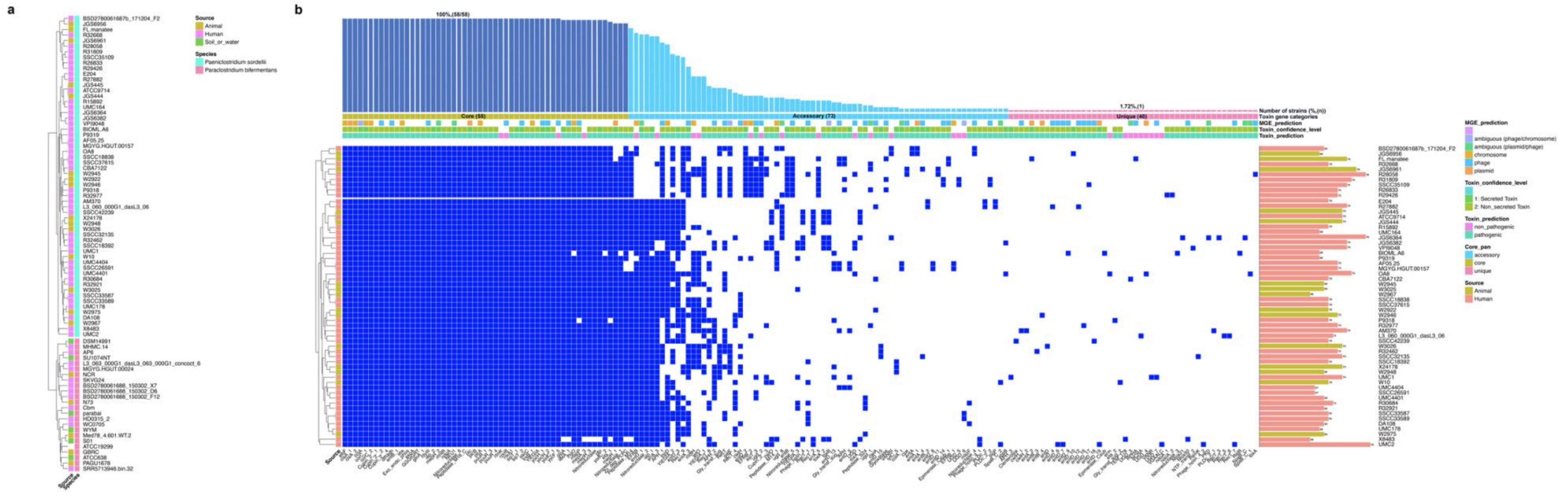

Figure S2 Differences in virulence factor coding capacity between *P. sordellii* and *P. bifermentans*. (a) Hierarchical clustering of *P. sordellii* and *P. bifermentans* using the pan-genomic virulence factor coding profiles. The coding gene count matrix was input as the data for clustering. (b) A presence-absence heatmap of virulence factor coding genes in the pangenome of *P. sordellii*.

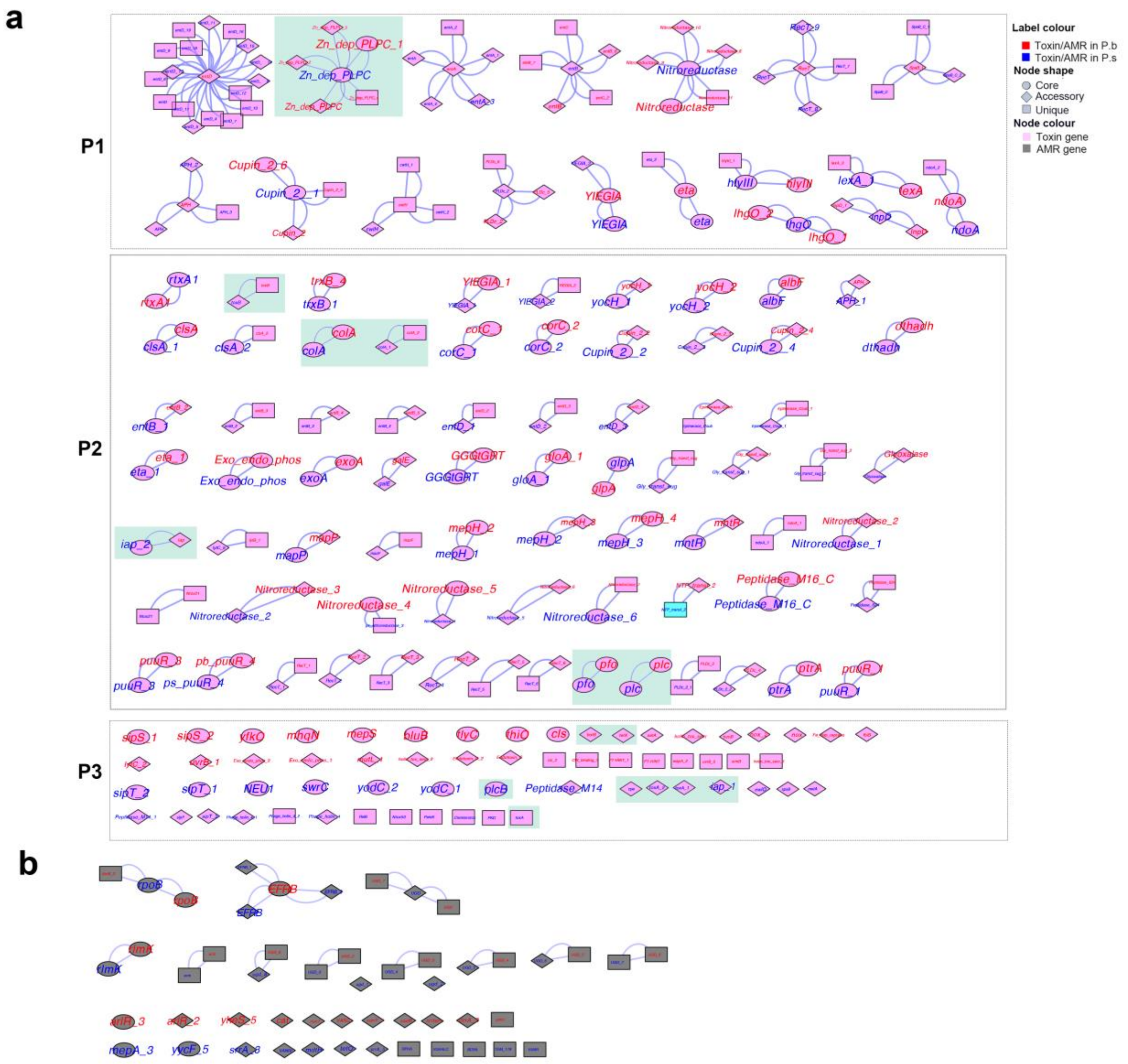

Figure S3 Global view of the virulence factor coding gene distributions in *P. bifermentans* and *P. sordellii*. The gene symbol size represents the counts of the genes in the pangenome of the species, and a bigger size represents higher counts. (a) Shared toxin coding genes between P.b and P.s. P1, P2 and P3 represent the shared toxin coding genes with more than two homologs, the shared toxin coding genes with only one homolog, and the unique toxin coding genes, respectively, between P.b and P.s. (b) The shared or unique AMR coding genes in each species.

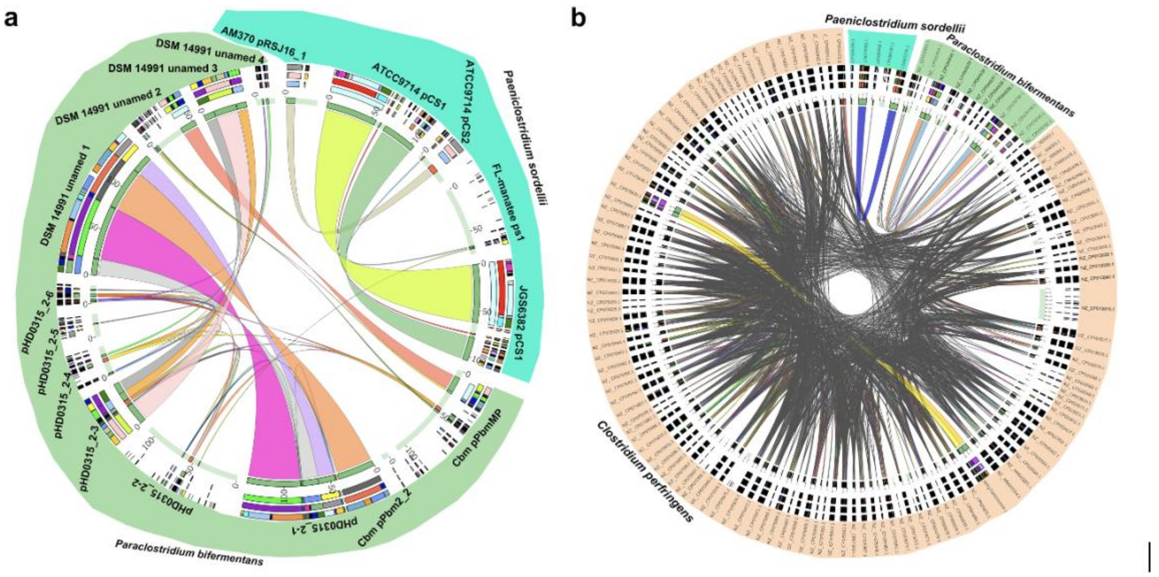

Figure S4 Plasmid synteny between the clostridial plasmids from *P. sordellii*, *P. bifermentans* and *C. perfringens*. Only plasmids documented in the NCBI Refseq database were retrieved and compared. (a) Plasmid synteny within the species *P. bifermentans*. (b) Plasmid synteny between *P. sordellii*, *P. bifermentans* and *C. perfringens*.

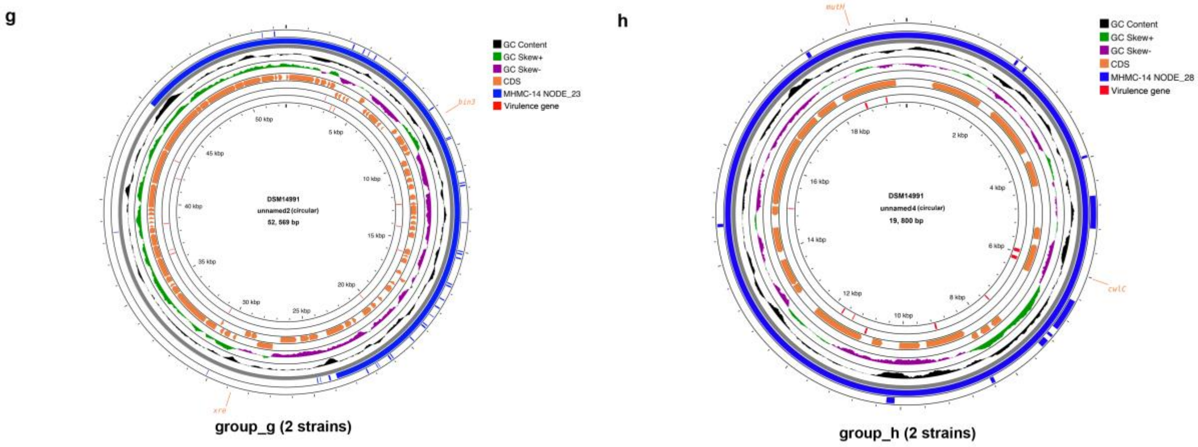

Figure S5 Representative plasmid groups in *P. bifermentans* and the coded functions. (g) group_g plasmids. (h) group_h plasmids.

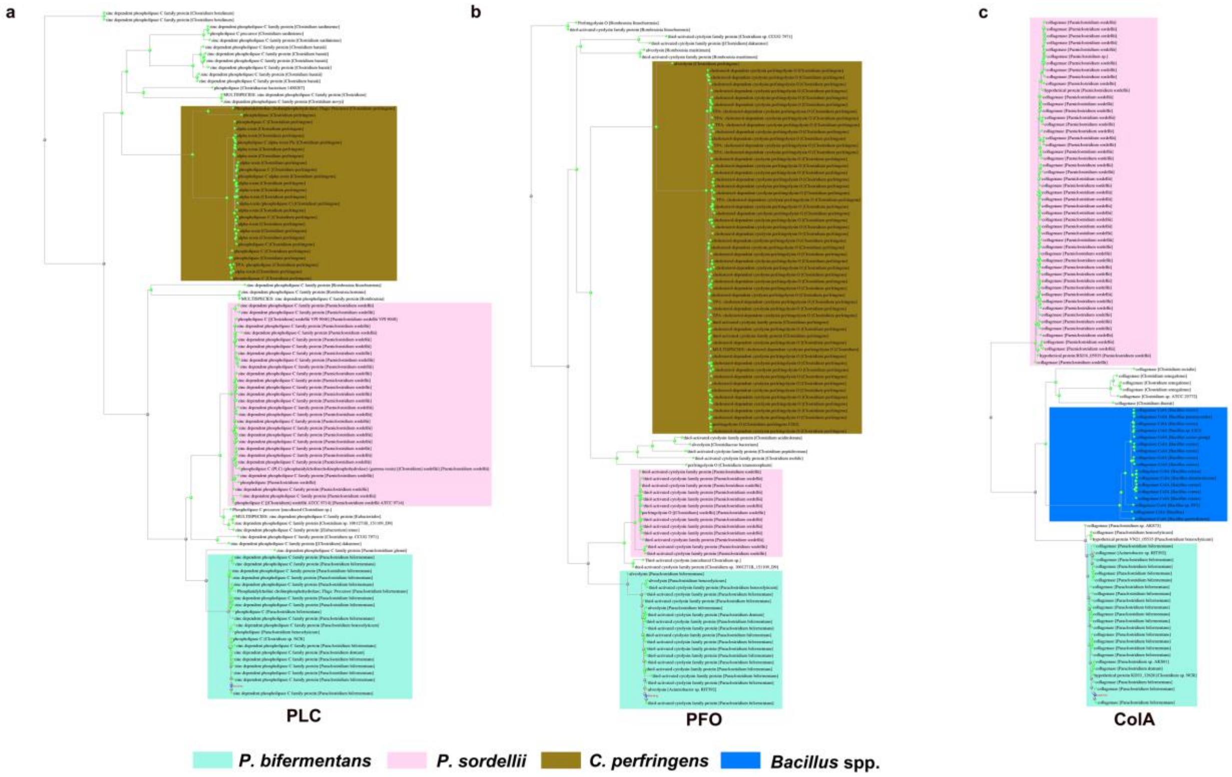

Figure S6 Distance tree of the three core toxin proteins constructed by aligning the representative sequences from *P. bifermentans* to the NCBI NR database using the default parameters of the BLASTP webservice. The tree was constructed using the fast minimum evolution method with default parameters. (a), (b), (c) represent the distance tree of PLC, PFO and ColA respectively.
